# Analysis of the predicting factors of recurrent wheezing in infants

**DOI:** 10.1186/s13052-019-0609-y

**Published:** 2019-01-29

**Authors:** Jia Zhai, Yingxue Zou, Jie Liu, Xingnan Jin, Cuian Ma, Jiao Li, Run Guo, Bing Huang

**Affiliations:** The second department of respiration, Tianjin Children’s Hospital, Tianjin, 300074 China

**Keywords:** Recurrent wheezing, Infants, Eczema, RSV infection

## Abstract

**Background:**

Clinically, asthma in children under 5 years old is under estimated because lack of diagnostic criteria. The current study was, therefore, designed to identify the predicting factors for recurrent wheezing in infants.

**Methods:**

One hundred forty-five infants under 3-year old hospitalized with respiratory diseases were enrolled into this study. Patients were followed up for one-year period after being discharged from the hospital and were, then, divided into recurrent wheezing group and non-recurrent wheezing group based on whether there was recurrent wheezing or not. Wheezing or recurrent wheezing was specifically monitored in addition to blood tests for allergic and respiratory diseases.

**Results:**

The prevalence of eczema and respiratory syncytial virus (RSV) infection were significantly higher in recurrent wheezing group than in control group (74.2% vs 45.8%; 32.3% vs. 13.3%, respectively, both *P* < 0.05); the percentage of blood eosinophil and serum eosinophil-derived neurotoxin (EDN) concentration at admission were also higher in recurrent wheezing group than in control group (3.10 ± 2.54% vs. 1.31 ± 1.15%; 68.67 ± 55.05 ng/mL vs. 27. 36 ± 19.51 ng/mL; respectively, both *P* < 0.001). Multivariate logistic regression analysis on eosinophil count and serum EDN concentration in predicting recurrent wheezing revealed that the eosinophil count showed the lowest sensitivity (51.6%) and highest specificity (90.4%), with the area under the ROC curve (AUC) of 0.752 ± 0.041; and that, in contrast, the serum EDN showed the highest sensitivity (88.7%) and lowest specificity (56.6%), with AUC of 0.795 ± 0.037.

**Conclusion:**

Combination of eosinophil count and serum EDN measurement may be better to predict the risk of recurrent wheezing in early life of childhood.

## Background

Wheezing in young children is a common clinical symptom of pediatric respiratory system disease, which is characterized by the expiratory phase from the chest with a continuous and sonorous voice, but sometimes also appear in the inspiratory phase, leading to increased breathing rate [1]. A prospective longitudinal observational study showed 26% of 6265 babies had at least one wheeze within 18 months after birth [2]. About half of children experience at least one wheeze before school age, and some of the infants with acute bronchiolitis can develop into recurrent wheezing [3]. Pellegrini-Belincho et al. [4] reported that probability of developing recurrent wheezing in the first year of life was significantly higher in the infants with a mother who has asthma, smoked but not consume a Mediterranean diet during pregnancy (79.7%) compared to the infants without of these factors (4.1%).

Respiratory tract virus infection, especially respiratory syncytial virus (RSV), may cause symptomatic wheezing and asthma exacerbations [5, 6]. Symptom of recurrent wheezing sometimes become exacerbated, called episodic viral wheeze (EVW) [7] or severe intermittent wheezing [8]. RSV is the most common cause of lower respiratory tract infections among infants and young children [9–12]. Studies have demonstrated that children hospitalized for RSV bronchiolitis during infancy were more likely to have subsequent episodes of wheezing and asthma during the first decade of life compared with children without RSV infection history [5, 6, 13].

Eosinophil contains cytotoxic granule proteins, such as eosinophil cationic protein (ECP), eosinophil peroxidase (EPO), and eosinophil-derived neurotoxin (EDN), they are all major basic proteins (MBP). EDN, a single-chain polypeptide with a molecular mass of 18.6 kDa, has been considered to be involved in the recurrent wheezing and asthma development in later life [14]. EDN expression is not restricted to eosinophil, as it is also detected in mononuclear cells and possibly neutrophils [15]. Each eosinophil expresses approximately 10 pg of EDN, with remarkable variation between individuals. On one hand, EDN is implicated in antiviral activity against respiratory infections [16]. On the other hand, EDN may also be associated with the pathogenesis of allergy, specifically in the respiratory tract, with the development of allergic asthma and recurrent wheezing [17].

Because of the complexity of recurrent wheeze in children, therapy choice for preventing severe exacerbation of asthma and episodic attack of wheezing is largely variable [18, 19]. In addition, while it is important for clinicians to predict recurrent wheezing to prevent severe episodic attack of wheezing, there are several biomarkers that can be used for predicting recurrent wheezing. In this regard, EDN might be one of the biomarkers for early identification of children at the highest risk of developing recurrent wheezing. The primary objective of this study was, therefore, to identify potential predicting factors from the parameters of laboratory tests for the recurrent wheezing in children through following up children with respiratory diseases.

## Methods

### Patients

Total 145 infants, who had various degree of wheezing and were hospitalized into the Internal Medicine Department, Tianjin Children’s Hospital from October 2015 to January 2016, were enrolled consecutively into this study. These patients were followed up for 1-year period after being discharged from the hospital. If the patient had at least 4 times wheezing within 1 year, it was defined as recurrent wheezing. Wheezing or recurrent wheezing was specifically monitored during the follow-up period.

Inclusion criteria: 1). Age was 3 years old or younger. 2). Patients were hospitalized with wheezing, which was detected by a physician through physical examination, and diagnosed as bronchiolitis, asthmatic bronchitis or Bronchial pneumonia. 3). Patients were not treated with steroid or leukotriene receptor antagonists 1 week before the enrollment.

Exclusion criteria: 1). Congestive deficient of bronchus and lung, primary or secondary airway stenosis, congestive heart disease, gastroesophageal reflux disease, bronchial foreign body. 2). Cancer or malignant diseases. 3). Primary or secondary immune deficiency or other immune-associated diseases. 4). Refuse to sign the consent or could not complete the follow-up examination due to either failed to show up or went to other hospitals.

### Data collection

1. General information: gender, age, dwelling area (urban or countryside), full-term or pre-term (< 37 weeks) delivery, vaginal delivery or C-section, first child or not, days of hospitalization, eczema, and drug allergy.
2. History of respiratory or other major disease, family history of asthma, allergic rhinitis or other allergic diseases.
3. Recurrent wheezing or not during the 1-year follow-up.

### Laboratory examination

Blood samples were collected at admission and the following laboratory tests were done in all of the patients: serum total Ig E, antigen-specific Ig E, food allergy Ig G, serum EDN, blood cell count, serum antibodies to the airway pathogens (influenza A, influenza B, para-influenza virus type 1–3, adenovirus, respiratory syncytial virus, mycoplasma, chlamydia pneumoniae, Q rickettsia, legionella pneumophila), virus in nasopharynx fluid including influenza A and B, adenovirus, respiratory syncytial virus, and para-influenza type 1–3.

Chest X-ray and CT, electrocardiogram, and echocardiography were also obtained in order to exclude potential causes of wheezing such as pneumonia, lung cysts, or tracheobronchomalacia.

### Statistical analysis

Data were analyzed with SAS 9.3 software. Distribution normality of continuous data was examined by Kolmogorov-Smirnov methods before the comparison. Data with normal distribution was expressed as mean ± SD and analyzed by *t* test. Continuous data with non-normal distribution was expressed as median and quartile (M, P25, P75) and analyzed with Wilcoxon two samples test. Categorical data were expressed as frequency and analyzed with Chi square examination. A receiver operating characteristic (ROC) curve was plotted with the predicting factors of recurrent wheezing and value of area under the ROC curve (AUC) as well as Somers’ D were calculated. Optimal cutoff point was applied when Yuden Index achieved maximum. Mann-Whitney method was used to compare variable parameters that predict area under the ROC. After establishing a model of logistic regression, stepwise regression was performed to screen potential risk factors for recurrent wheezing. *P* < 0.05 was considered as significant.

## Results

### General characteristics of the infants

Total 62 infants (male 42 and female 20, median age of 5 months) with recurrent wheezing (as recurrent whoop group) were hospitalized for a median of 6 (4,11) days, 40 of whom were living in urban; while 83 infants (male 53 and female 30, median age of 4 months) without recurrent wheezing (as control group) were hospitalized for a median of 5 (4,10) days, 52 of whom were living in urban (Tables 1 and 2). Twenty-eight out of the 62 infants with recurrent wheezing were the first child in their family, 35 were vaginal delivery and 27 were in C-section. Seventy-one of 83 infants without recurrent wheezing were the first child in their family and all 83 infants were vaginal delivery.

**Table 1:** Patients’ demographic characteristics

	Without Recurrent Wheezing (*n* = 83)	With Recurrent Wheezing (*n* = 62)	*P*
Age (month), median (quartiles)	4 (2, 8)	5 (2, 9)	0.809
Sex, N (%)			0.626
Male	53 (63.9)	42 (67.7)	
Female	30 (36.1)	20 (32.3)	
Dwelling, N (%)			0.817
Urban	52 (62.7)	40 (64.5)	
Countryside	31 (37.4)	22 (35.5)	
Delivery, N (%)			0.259
C-section	44 (53.0)	27 (43.6)	
Vaginal	39 (46.9)	35 (56.5)	
Family history, N (%)	10 (12.1)	11 (17.7)	0.335
First child, N (%)	36 (43.4)	28 (45.2)	0.830
Term birth, N (%)	71 (85.5)	58 (93.6)	0.128
Eczema, N (%)	38 (45.8)	46 (74.2)	0.001
Drug allergy, N (%)	2 (2.5)	3 (4.8)	0.782

**Table 2:** Comparison of the pathogens

	Without Recurrent Wheezing (*n* = 83)	With Recurrent Wheezing (*n* = 62)	*P*
History (day), median (quartiles)	5 (4, 10)	6 (4, 11)	0.592
Fever (day), median (quartiles)	0 (0, 1)	0 (0, 1)	0.983
Pathogen infection, N (%)	22 (26.5)	28 (45.2)	0.019
RSV infection, N (%)	11 (13.3)	20 (32.3)	0.006
Mycoplasma infection, N (%)	5 (6.0)	5 (8.1)	0.882
Hib infection, N (%)	2 (2.4)	1 (1.6)	1.000
Influenza B infection, N (%)	1 (1.2)	4 (6.5)	0.087

Interestingly, prevalence of eczema was significantly higher in the infants with recurrent wheezing compared with those without recurrent wheezing (74.2% vs. 45.8%, *P* < 0.05). However, there was no significant difference in drug allergy between the two groups (4.8% with vs 2.5% without recurrent wheezing, *P* > 0.05).

### Comparison of the pathogens and other laboratory examination

Pathogens from the patients’ up airway fluid were examined. The prevalence of various virus infection was significantly higher in the infants with recurrent wheezing compared with those without recurrent wheezing (28/62, 45.2% vs. 22/83, 26.5%, *P* < 0.05). Specifically, prevalence of respiratory syncytial virus (RSV) infection was significantly higher in the infants with recurrent wheezing compared with the infants without recurrent wheezing (20/62, 32.3% vs. 11/83, 13.3%, *P* < 0.05). Differences of other pathogens including mycoplasma and influenza virus infection were not significant (Table 2).

The infants with recurrent wheezing showed significantly higher number of eosinophil and concentration of eosinophil-derived neurotoxin (EDN) compared with the infants without recurrent wheezing (eosinophil: 3.10 ± 2.54% vs 1.31 ± 1.15%, *P* < 0.001; EDN: 68.67 ± 55.05 vs 27. 36 ± 19.51 ng/mL, *P* < 0.001). In addition, C-reactive protein (CRP), ferritin, white cell count (WBC), lactate dehydrogenase (LDH), procalcitonin (PCT), and γ-globin were slightly, but not significantly, higher in the infants with recurrent wheezing than those in the infants without recurrent wheezing (Table 3). Interestingly, serum Ig E was slightly but not significantly lower in the infants with recurrent wheezing compared with that in the infants without recurrent wheezing (43.71 ± 80.44 IU/mL vs. 44.58 ± 92.23 IU/mL). In addition, there was no significant difference between the two groups in terms of received treatment including antibiotics, intravenous immunoglobulin (IVIG), and steroid therapy (Table 4).

**Table 3:** Results of laboratory examination

	Without Recurrent Wheezing (*n* = 83)	With Recurrent Wheezing (*n* = 62)	*P*
Ig E positive to antigen, N (%)	17 (20.48)	8 (12.90)	0.232
Ig G positive to antigen, N (%)	44 (53.01)	32 (51.61)	0.867
Serum Ig E (IU/mL), mean ± SD	44.58 ± 92.24	43.71 ± 80.44	0.978
CRP(mg/L), median (quartiles)	1 (1, 1)	1 (1, 4)	0.168
Ferritin (μg/L), mean ± SD	136.39 ± 199.22	164.77 ± 160.97	0.088
Hb (g/L), mean ± SD	115.02 ± 13.44	113.26 ± 13.39	0.434
WBC (×10^9^/L), median (quartiles)	8.83 (6.99, 10.31)	8.53 (6.63, 10.13)	0.678
N (%), median (quartiles)	28 (20, 39)	25 (19, 34)	0.183
LDH (U/L), median (quartiles)	436 (355, 555)	408.5 (334, 585)	0.767
La (mmol/L), median (quartiles)	3.49 (2.46, 4.35)	3.22 (2.55, 4.16)	0.520
PCT (ng/L), median (quartiles)	0.08 (0.05, 0.1)	0.08 (0.06, 0.1)	0.116
γ-globin (g/L), mean ± SD	7.21 ± 2.63	7.29 ± 2.31	0.646
Eosinophil (%), mean ± SD	1.31 ± 1.15	3.10 ± 2.54	< 0.001
EDN (ng/mL), mean ± SD	27.36 ± 19.51	68.67 ± 55.05	< 0.001

**Table 4:** Treatment of the patients

	Without Recurrent Wheezing (*n* = 83)	With Recurrent Wheezing (*n* = 62)	*P*
Antibiotics, N (%)			0.244
Yes	57 (68.67)	48 (77.42)	
No	26 (31.33)	14 (22.58)	
IVIG Therapy *, N (%)			0.090
Yes	11 (13.25)	3 (4.84)	
No	72 (86.75)	59 (95.16)	
Systemic steroid, N (%)			0.100
Yes	23 (27.71)	10 (16.13)	
No	60 (72.29)	52 (83.87)	

*IVIG: intravenous immunoglobulin

### Risk factors of recurrent wheezing and their prognostic value

As shown in Table 5, by stepwise regression analysis, the following factors were identified as potential risk factors for recurrent wheezing: eosinophil with OR (95% CI) of 2.137 (1.507, 3.03), *P* < 0.001; EDN with OR (95% CI) of 1.040 (1.021, 1.059), *P* < 0.001; eczema with OR (95% CI) of 4.313 (1.608, 11.571), *P* = 0.004; and RSV infection with OR (95% CI) of 3.481 (1.143, 10.605), *P* < 0.028.

**Table 5:** Potential risk factors of recurrent

Independent Variable	Parameter value^*^	SD	OR (95% CI)	*P*
Constant term	−3.244	0.599	–	< 0.001
Eosinophil	0.759	0.178	2.137 (1.507, 3.030)	< 0.001
EDN	0.039	0.009	1.040 (1.021, 1.059)	< 0.001
Eczema	0.731	0.252	4.313 (1.608, 11.571)	0.004
RSV infection	0.624	0.284	3.481 (1.143,10.605)	0.028

^*^Logistic multivariable regression: independent variables were the parameters shown in Tables 1 and 4 with *P* < 0.01, and “recurrent” was the dependent value. Stepwise regression was performed to screen potential risk factors. *P* value for inclusion or exclusion of the independent variables was 0.1

### Multivariate logistic regression analysis and diagnostic value of eosinophil count and serum EDN level

**Fig. 1:**
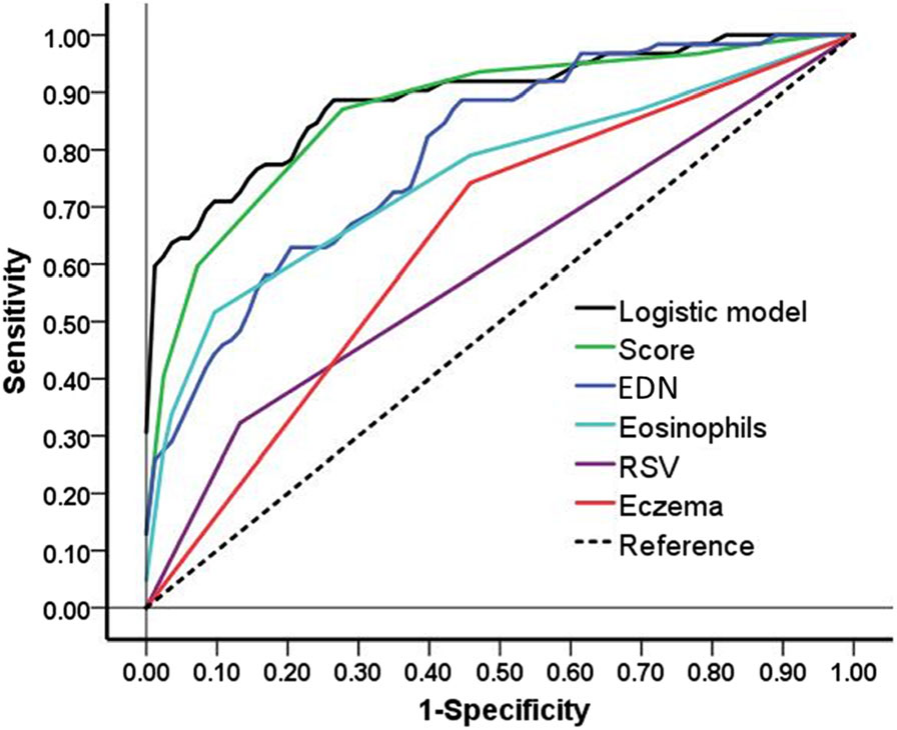
Receiver operating characteristic (ROC) analysis of the sensitivity and specificity for the prediction of recurrent wheezing by logistic model, score, EDN, Eosinophils RSV, and eczema. Vertical axis: sensitivity; horizontal axis: specificity

**Fig. 2:**
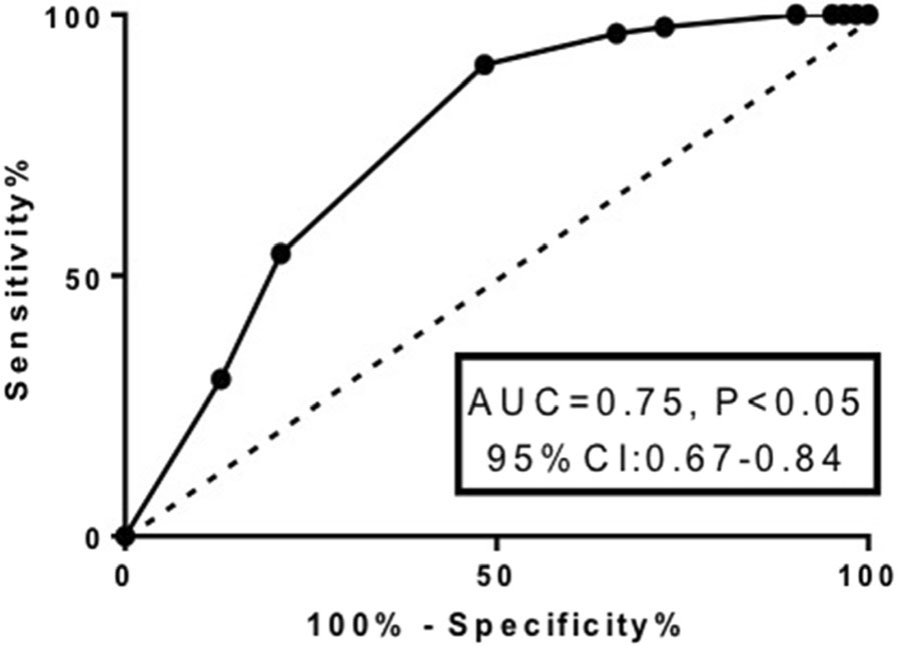
ROC analysis of the sensitivity and specificity for the prediction of recurrent wheezing by eosinophil count. Vertical axis: sensitivity; horizontal axis: specificity

**Fig. 3:**
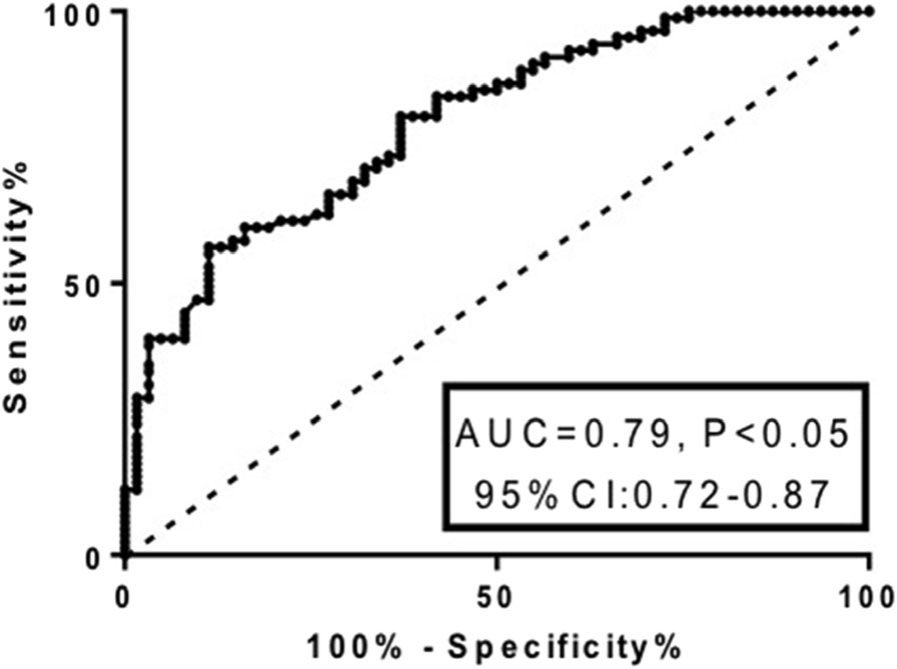
ROC analysis of the sensitivity and specificity for the prediction of recurrent wheezing by serum EDN level. Vertical axis: sensitivity; horizontal axis: specificity

The value of the risk factors to predict recurrent wheezing was further analyzed by logistic multivariable regression and by receiver operating characteristic (ROC) curve. As presented in Fig. 1 and Table 6, of the evaluated parameters, sensitivity and specificity of eosinophil count and serum EDN possessed stronger power for the prognostic evaluation of recurrent wheezing, which was consistent with the results that eosinophil count and serum EDN were significantly higher in the infants with recurrent wheezing. Specifically, the sensitivity of eosinophil count was 51.6% and specificity of it was 90.4%, the area under the ROC curve (AUC) = 0.752 ± 0.041, and the cutoff value was 2.50% (Fig. 2 and Table 6). Similarly, sensitivity of the serum EDN for the diagnosis of recurrent wheezing was 88.7%, specificity of it was 56.6%, AUC = 0.795 ± 0.037, and the cutoff value was 20.18 ng/mL (Fig. 3 and Table 6). Furthermore, comparison of the risk factor scores in predicting recurrent wheezing indicated that RSV infection was highest in score (AUC value ±SD: − 0.274 ± 0.045, *P* < 0.001, Table 7) followed by eczema (AUC value ±SD: − 0.227 ± 0.046, *P* < 0.001, Table 7), eosinophil count (AUC value ±SD: − 0.117 ± 0.033, *P* < 0.001, Table 7), and EDN (AUC value ±SD: − 0.074 ± 0.029, *P* = 0.01, Table 7).

**Table 6:** Logistic multivariable regression and prognostic value of the risk factors

Source	AUC ± SD	95% CI of AUC		Somers’ D	Cutoff	Sensitivity	Specificity
		Lower limit	Upper limit				
Logistic Model	0.892 ± 0.028	0.837	0.946	0.783	–	–	–
Eosinophil	0.752 ± 0.041	0.671	0.833	0.504	2.5	0.516	0.904
EDN	0.795 ± 0.037	0.723	0.866	0.590	20.18	0.887	0.566
Eczema	0.642 ± 0.039	0.565	0.719	0.284	–	–	–
RSV	0.595 ± 0.035	0.526	0.664	0.190	–	–	–
Scoring Model	0.869 ± 0.030	0.809	0.928	0.737	5.5	0.871	0.723

**Table 7:** Comparison of Scoring and Logistic models in evaluating prognostic factors

Comparison	AUC value ± SD	95%CI of AUC		*P*
		Lower limit	Upper limit	
Logistic model - Score	0.023 ± 0.016	−0.008	0.054	0.149
Eosinophil - Score	−0.117 ± 0.033	−0.181	− 0.053	< 0.001
EDN - Score	−0.074 ± 0.029	−0.130	− 0.017	0.010
Eczema - Score	−0.227 ± 0.046	−0.318	− 0.136	< 0.001
RSV Infection - Score	−0.274 ± 0.045	−0.361	− 0.186	< 0.001

## Discussion

Recurrent wheezing and/or asthma are common chronic respiratory disease in children. In the current study, children at age of 3 years old or younger, who were hospitalized with wheezing, were followed-up for 1 year to analyze factors that may predict recurrent wheezing. Our results showed the four parameters of eczema, RSV infection, eosinophil count and EDN were all significantly higher in infants with recurrent wheezing than that in infants without recurrent wheezing, strongly suggesting they were the risk factors related to recurrent wheezing. Furthermore, EDN reached a better AUC of 0.795 and 0.752, even though the sensitivity of eosinophil count and the specificity of EDN were only 0.516 and 0.566, respectively. Thus, the combination of eosinophil count and serum EDN quantification may be served as one of the biomarkers to predict the recurrent wheezing in clinical practice.

Several recent studies have reported that respiratory infections in infants or early in life may have profound impact for the development of respiratory diseases including wheezing and/or asthma [6, 20, 21]. In this regard, studies have highlighted that RSV-related respiratory infection in preterm infants may be associated with an increased prevalence of early childhood wheezing and asthma in later adulthood [5, 13, 22, 23]. A study reported the prophylaxis effect of palivizumab, a humanized monoclonal antibody targeting RSV, on 444 preterm infants and found that prevalence of atopic asthma during 5 years of life was not different between the groups of infants with or without palivizumab treatment [18]. However, physician-diagnosed recurrent wheezing was observed in 15.3 and 31.6% (*P* = 0.003) of the treated and untreated groups, respectively [24]. Consistent with the previous studies, we found that incidence of RSV infection was significantly higher in the infants with recurrent wheezing compared with the infants without recurrent wheezing.

Allergic diseases may share common mechanisms in the pathogenesis [25, 26]. In this regard, eosinophil plays a key role in the pathogenesis and development of atopic disease including asthma and eczema [14, 27]. Eosinophil secretes cytotoxic granules including major basic protein (MBP), eosinophil cationic protein (ECP), eosinophil peroxidase (EPO), and eosinophil-derived neurotoxin (EDN) [14]. Eosinophil granules contain a crystalloid core composed of MBP-1 and a matrix composed of ECP, EDN, and EPO [27]. MBP, EPO and ECP are toxic to a variety of cells and tissues, including heart, brain, and bronchial epithelium [28, 29]. EDN is released by activated eosinophil following infection, allergy or both [30]. A study on the serum EDN level and recurrent wheezing episodes for 12 months in the 6–12 months old infants who had RSV bronchiolitis indicated that 3-month serum EDN levels correlated significantly with the total number of wheezing episodes at 12 months in both groups of treated with placebo or leukotriene receptor antagonist [31, 32]. Consistently, the current study demonstrated that EDN level was significantly higher in the infants with recurrent wheezing and that serum EDN had stronger power for the prognostic evaluation of recurrent wheezing.

Eczema is a very common skin chronic inflammation in infancy, characterized by redness, itchiness and/or dryness. Although the infants with eczema may more frequently develop into allergies and asthma, correlation of eczema and recurrent wheezing in infants has not been thoroughly studied. It was found that among children within 1-year-old age, the correlation between allergy and eczema was strongest, and the correlation between allergy and asthma and rhinitis began at age 3 and gradually lasted until age 16 [33]. Zinelli et al. reported that forced expiration of nitric oxide (FE(NO)) was increased in children with atopic eczema compared with that in healthy children, even in the absence of respiratory symptoms and in the presence of normal lung function [34], and thus, they proposed that FE(NO) may be a predictive biomarker for the development of asthma in children. In our study, the prevalence rate of eczema was significantly higher in recurrent wheezing group than in control group, and eczema was the 2nd potential factor in predicting recurrent wheezing by comparison of risk factor scores. Consistently, a logistic regression analysis revealed with the 3 independent risk factors (history of eczema, food allergies, and history of parent rhinitis) for children’s sustained breathing, only the history of eczema was the main risk factor related with sustained whoop [35].

We realized there were some limitations in the study. Firstly, the potential effect of environmental factors, such as parental smoking and air pollution, on recurrent wheezing was not assessed. In this regard, recent longitudinal studies have investigated potential associations of the onset of childhood wheezing illnesses with prenatal exposures including parental smoking, outdoor air pollution, and maternal stress on childhood wheezing illnesses [36–38]. In a birth cohort from the Netherlands, the authors found ~ 50% increased odds of persistent wheeze and ~ 65% increased odds of asthma at age 6 years in children whose mothers smoked at least five cigarettes per day through the pregnancy [36]. A nested case-control study [39] in Canada found that prenatal exposure to higher levels of nitrogen monoxide, nitrogen dioxide, carbon monoxide, sulfur dioxide, and particular matter of 10 μm or less (PM_10_) increased the risk of asthma in preschoolers, particularly in low birth-weight infants. Secondly, atopic sensitization to aeroallergens is well-recognized as a risk factor for the subsequent development of recurrent wheeze (and asthma) [40]. Status of atopic sensitization to allergens, however, was not assessed in the current study.

## Conclusions

The current study assessed potential predicting factors for the recurrent wheezing in children 3 years old or younger. The prevalence of respiratory syncytial virus (RSV) infection, the eosinophil percentage and serum level of eosinophil-derived neurotoxin (EDN) were the predominant risk factors and could be used to predict recurrent wheezing in infants. Of the three factors, eosinophil count showed the lowest sensitivity but highest specificity, and in contrast, the serum EDN showed the highest sensitivity and lowest specificity. Thus, combination of eosinophil count and serum EDN measurement may be better to predict the risk of recurrent wheezing in early life of childhood.

## Abbreviations

ECP: Eosinophil cationic protein
EDN: Eosinophil-derived neurotoxin
EVW: Episodic viral wheeze
HRV: Human rhinovirus
MBP: Major basic proteins
ROC: Receiver operating characteristic
RSV: Respiratory syncytial virus
